# Pharmacokinetic interactions between artesunate-mefloquine and ritonavir-boosted lopinavir in healthy Thai adults

**DOI:** 10.1186/s12936-015-0916-8

**Published:** 2015-10-09

**Authors:** Siwalee Rattanapunya, Tim R. Cressey, Ronnatrai Rueangweerayut, Yardpiroon Tawon, Panida Kongjam, Kesara Na-Bangchang

**Affiliations:** Faculty of Science and Technology, Chiang Mai Rajabhat University, Chaing Mai, Thailand; Department of Medical Technology, Faculty of Associated Medical Sciences, Programme for HIV Prevention and Treatment (PHPT/IRD URI 174), Chiang Mai University, Chiang Mai, Thailand; Harvard School of Public Health, Boston, MA USA; Mae Sot General Hospital, Mae Sot, Tak Province Thailand; Centre of Excellence in Pharmacology and Molecular Biology of Malaria and Cholangiocarcinoma, Graduate Programme in Bioclinical Sciences, Chulabhorn International College of Medicine, Thammasat University, Pathumthani, 12121 Thailand

## Abstract

**Background:**

Concomitant use of anti-malarial and antiretroviral drugs is increasingly frequent in malaria and HIV endemic regions. The aim of the study was to investigate the pharmacokinetic interaction between the anti-malarial drugs, artesunate-mefloquine and the antiretroviral drug, lopinavir boosted with ritonavir (LPV/r).

**Methods:**

The study was an open-label, three-way, sequential, cross-over, pharmacokinetic study in healthy Thai adults. Subjects received the following treatments: Period 1: standard 3-day artesunate-mefloquine combination; Period 2 (2 months wash-out): oral LPV/r 400 mg/100 mg twice a day for 14 days; and, Period 3: artesunate-mefloquine and LPV/r twice a day for 3 days. Sixteen subjects (eight females) were enrolled and pharmacokinetic parameters were determined by non-compartmental analysis.

**Results:**

In the presence of LPV/r, artesunate C_max_ and systemic exposure were significantly increased by 45–80 %, while the metabolic ratio of dihydroartemisinin to artesunate was significantly reduced by 72 %. In addition, mefloquine C_max_ and systemic exposure were significantly reduced by 19–37 %. In the presence of artesunate-mefloquine, lopinavir C_max_ was significantly reduced by 22 % but without significant change in systemic drug exposure. The 90 % CI of the geometric mean ratio (GMR) of AUC_0−∞_ and C_max_ were outside the acceptable bioequivalent range for each drug. Drug treatments were generally well tolerated with no serious adverse events. Vertigo, nausea and vomiting were the most common adverse events reported.

**Conclusion:**

The reduction in systemic exposure of all investigated drugs raises concerns of an increased risk of treatment failure rate in co-infected patients and should be further investigated.

## Background

Malaria and human immunodeficiency virus (HIV) infections remain major global health burdens [1]. In 2012, there was an estimated 207 million cases of malaria worldwide, leading to 627,000 deaths [2]. It was estimated that 35 million people were living with human immunodeficiency virus (HIV) in 2014 and despite significant improvements in HIV prevention and treatment, there were also 2.1 million new infections and 1.5 million HIV-related deaths worldwide [3]. Management of malaria and HIV co-infection is challenging with possible adverse pathological, clinical, pharmacological, and epidemiological interactions between malaria and HIV infections and treatments [4–12]. Artemisinin-based combination therapy (ACT) is recommended by the World Health Organization (WHO) as first-line treatment for acute, uncomplicated *Plasmodium falciparum* malaria [13]. A 3-day course of artesunate-mefloquine combination therapy is commonly used in Southeast Asia to cope with multidrug-resistant *P. falciparum* [13]. Artesunate is responsible for the initial rapid decline in parasites, while mefloquine persists in the body much longer than artesunate to kill the remaining parasites [13]. For HIV therapy, ritonavir-boosted protease inhibitors (PIs) are currently recommended by WHO as part of second-line antiretroviral therapy for adults. Globally, lopinavir/ritonavir (LPV/r) remains the most commonly used PI due to its availability as a fixed-dose combination and high genetic barrier to resistance [14].

Artesunate is primarily metabolized via esterase-mediated hydrolysis and cytochrome P450 (CYP) 2A6 enzyme to the active metabolite dihydroartemisinin [15]. Dihydroartemisinin is subsequently metabolized via uridinediphosphate glucuronosyltransferases (UGTs) 1A8/9 and 2B7 and excreted in the bile [16]. Biotransformation of its combination partner mefloquine and LPV/r is via CYP3A4 [17–21]. Ritonavir is a potent inhibitor and/or inducer of CYP3A4 and several CYP3A4, CYP2B6 and CYP2D6 activities [22–25] and is a substrate for several membrane transporter proteins [24, 26]. The potential for pharmacokinetic drug interactions between ACT, notably artemether-lumefantrine and LPV/r has been documented [27]. The aim of the current study was to investigate the pharmacokinetic interactions between artesunate-mefloquine and LPV/r when given together in healthy Thai adults.

## Methods

### Subjects and study design

This was an open-label, three-way, sequential, cross-over, pharmacokinetic study in healthy adult volunteers. Inclusion criteria included: (1) males and non-pregnant females, (2) aged 15–55 years, (3) body weight 40–65 kg, (4) non-smokers and non-alcohol drinkers, and, (5) residents of Mae Sot district, Tak Province. Exclusion criteria were those with: (1) hepatic or renal diseases, (2) history of using any drug or herbal medicine within the past 14 days, except antipyretic or anti-emetic drugs, or, (3) history of intolerance to artesunate, mefloquine, lopinavir, and ritonavir. Written informed consent for study participation was obtained from each subject before study. The minimum requirement of the sample size for the study was 16 subjects based on a = 0.05, target power = 80 % (b = 0.02) and CV (coefficient of variation) of clearance of artesunate (the most variable drug) = 20 %. Consenting adults were screened for eligibility and a physical examination, electrocardiogram (ECG), and laboratory safety tests (haematology, biochemistry, urinalysis, and pregnancy status) were performed.

The study protocol was approved by the Institute for Development of Human Research Protection (IHRP) at the Ministry of Public Health in Thailand. Study procedures were conducted in accordance with the Declaration of Helsinki and national and institutional standards.

### Drug administration

**Fig. 1:**
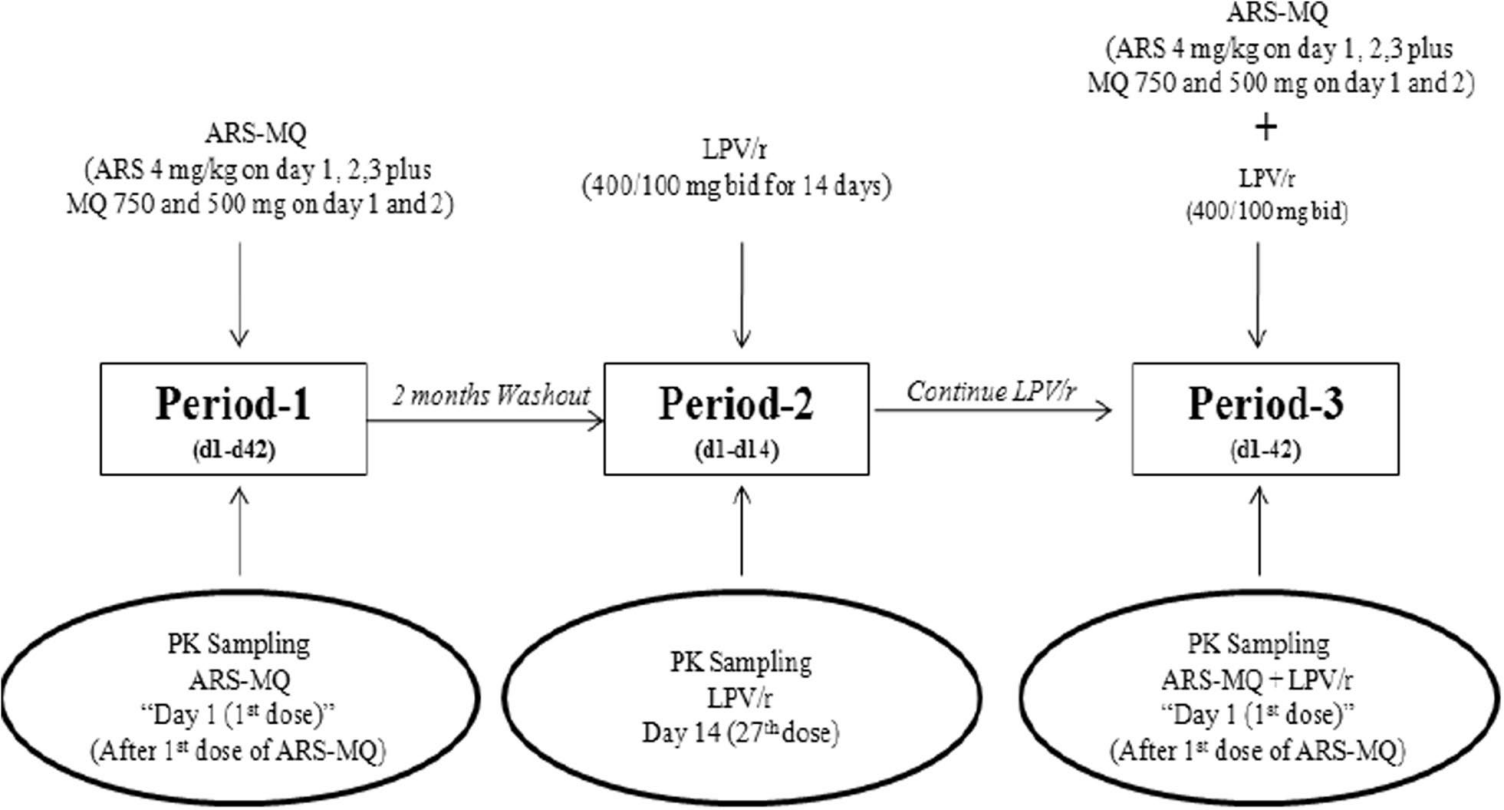
Schematic diagram depicting the study design for investigation of pharmacokinetic interaction between a 3-day artesunate-mefloquine (ARS-MQ) and lopinavir-boosted with ritonavir (LPV/r) in healthy Thai subjects

Figure 1 summarizes the study design. The pharmacokinetic sampling was performed sequentially on three occasions. Period 1: starting on Day 1, subjects received a 3-day course of oral artesunate-mefloquine (artesunate 200 mg on Days 1, 2, and 3 plus mefloquine 750 and 500 mg on Days 1 and 2, respectively). Artesunate doses were given as four tablets (50 mg artesunate per tablet, manufactured by Guilin Pharmaceutical Co. Ltd., China). Mefloquine doses were given as three tablets on Day 1 and two tablets on Day 2 (250 mg mefloquine per tablet, manufactured by Atlantic Pharmaceutical Ltd., China). There was a 2-months wash-out period between Period 1 and Period 2. Period 2: subjects received oral doses of LPV/r (400/100 mg), twice daily, for 14 days (27 doses). There was no washout between Period 2 and Period 3. Period 3: subjects received the same 3-day oral artesunate-mefloquine combination as in Period 1 in combination with LPV/r 400/100 mg, twice a day for 3 days.

All subjects were admitted to Mae Sot General Hospital for observation during the pharmacokinetic sampling period and drug dosage was taken at least 2 h before meal with water (standard volume 150 mL). All drug doses were administered under supervision of the investigator team. Only analgesics/antipyretics (paracetamol) and anti-emetics (dimenhydrinate) were allowed in cases of fever and nausea. Drugs with potential interactions with the study drugs, i.e., inhibitors and inducers of CYP3A4 and CYP2A6 were disallowed during the study period [28, 29].

### Assessments of safety and tolerability

Safety and tolerability of the drugs were assessed based on clinical and laboratory assessments during follow-up (42 days after Period 1 and 3, and 14 days after Period 2 according to NIH/NCI common toxicity criteria (CTC) grading system for adverse events [30]. Clinical assessments included physical examination and monitoring of vital signs and adverse events. Safety laboratory assessments (haematology, biochemistry and urinalysis) were performed during each period. All female subjects had a pregnancy test (b-human chorionic gonadotropin test) performed during each study period. Any abnormal laboratory result was followed up with repeat checks every week until it returned to normal.

### Blood sample collection for pharmacokinetic assessment

During Period 1 (3-day artesunate-mefloquine alone) and Period 3 (3-day artesunate-mefloquine plus LPV/r), blood samples (3 mL each) for determination of mefloquine, artesunate and dihydroartemisinin concentrations were drawn at pre-dose (before the first dose), and at one, two, four, six, eight, 12, 24, 25, 30, 36, 48, and 49 h, and seven, 14, 21, 28, 35, and 42 days post-dose (after the first dose). During Period 2 and Period 3, blood sample (3 mL each) for determination of lopinavir and ritonavir concentrations were drawn pre-dose (27th dose), and at one, two, four, six, eight, and 12 h post-dose. Immediately after collection, all blood samples were centrifuged (1200×*g*, 10 min) and the plasma samples were stored at −80 °C until analysis.

### Measurement of drug concentrations

Analysis of plasma artesunate/dihydroartemisinin and mefloquine concentrations was performed at the Centre of Excellence in Pharmacology and Molecular Biology of Malaria and Cholangiocarcinoma, Thammasat University. Measurement of plasma concentrations of artesunate and its active plasma metabolite dihydroartemisinin were performed using liquid chromatography mass-spectrometry (LC–MS/MS), according to the methods of Thuy et al. and Lindegardh et al. with modifications [31, 32]. An Agilent 1260 LC system (Agilent Technologies, CA, USA) coupled with an API 5000 triple quadrupole mass spectrometer (Applied Biosystems/MDS SCIEX, Foster City, USA), with a TurboVTM ionization source (TIS) interface operated in the positive ion mode, was used for the multiple reaction monitoring LC–MS/MS analysis. TIS temperature was maintained at 500 °C and the TIS voltage was set at 5500 V. Nitrogen gas was supplied from an AB-3G (Peak Scientific, Inchinnan, UK). The curtain, nebulizer (GS1) and TIS (GS2) gases were set at 25, 45 and 50 psi, respectively. Quantification was performed using selected reaction monitoring for the transitions *m/z* 402 → 267 (artesunate), 302 → 163 (dihydroartemisinin), and 300 → 209 (the internal standard artemisinin). Chromatographic separation was performed using an Elipse XDB C18 column (5 µm, 4.6 mm × 150 mm; Thermo Fisher Scientific, MA, USA) protected with an Eclipse XDB-C8 guard column (Thermo Electron Corporation, MA, USA). The gradient mobile phase consisted of a mixture of 10 mM ammonium acetate with 0.1 % glacial acetic acid and acetonitrile running at a flow rate of 0.7 mL/min. Total run time was 12 min. The limit of quantification (LOQ) for artesunate and dihydroartemisinin were 1 and 2 ng/mL, respectively. Recoveries for both compounds were between 82–92 and 81–98 %, respectively. The intra- and inter-day coefficients of variation (% CV) of artesunate *vs* dihydroartemisinin were 0.7–4.7 vs 4.5–7.3 %; and 3.9–18.3 vs 4.9–23.1 %, respectively.

Measurement of plasma concentrations of mefloquine was performed using high performance liquid chromatography (HPLC) according to the method of Karbwang et al. [33] with modifications. Chromatographic separation was performed on a Hypersil ODS column (5 µm, 4.6 mm × 250 mm; Thermo Fisher Scientific, MA, USA) protected with an Eclipse XDB-C8 guard column (Thermo Electron Corporation, MA, USA). The mobile phase consisted of a mixture of phosphate buffer (adjusted to pH 2.8 with 1 M phosphoric acid), acetonitrile and methanol at a ratio of 40:30:30 % (v:v:v), running at the flow rate of 1.0 mL/min. The UV detector was set at the wavelength of 220 nm. The assay LOQ was 2.5 ng/mL. Recovery varied between 83 and 94 %. The intra- and inter-day CV of mefloquine were 1.2–6.2 and 4.3–14.5 %, respectively.

Lopinavir and ritonavir plasma drug concentrations were measured using a validated HPLC assay at the PHPT Pharmacology Laboratory, Faculty of Associated Medical Sciences, Chiang Mai University [34]. The assay LOQ for lopinavir and ritonavir were 100 and 50 ng/mL, respectively. The recoveries of lopinavir and ritonavir were between 96–112 and 90–94 %, respectively. Intra- and inter-assay precisions were less than 4 % of the coefficient of variation. This laboratory participates in the NIAID Clinical Pharmacology Quality Assurance Programme Proficiency Testing [35].

Quality control (QC) samples for all drugs/metabolite under investigation were run in duplicate in each analytical batch at low, medium and high concentrations. Criteria for acceptability were four out of six of the QC analyses to lie inside 100 ± 15 % of the nominal values.

### Pharmacokinetic analysis

Pharmacokinetic parameters were determined using a non-compartmental analysis using WinNonLin software (version 6.3, Pharsights, Certara, USA). Concentrations of drugs lower than the LOQ levels were expressed as zero (undetectable). The C_max_ (maximum concentration); t_max_ (time of maximum concentration) were determined by direct inspection of the plasma concentration–time data. AUC (area under the concentration–time curve) from time 0 to 12 (AUC_0−12h_), 0 to 24 (AUC_0−24h_), 0 to 48 (AUC_0−48h_), 0 to 168 (AUC_0−168h_) h, and total AUC (AUC_0−∞_) were calculated using the trapezoidal rule. The extrapolated AUC from the last sampling time to infinity was estimated from Ct/elimination rate constant (l_z_). l_z_ was calculated from at least five concentration–time points of elimination phase. Apparent oral clearance (CL/F) was calculated as dose/AUC_0−∞_. Volume of distribution (V_z_/F) was calculated as CL/F/l_z_, and the terminal half-life (t_1/2z_) was calculated as 0.693/l_z_. Metabolic ratio (MR) was defined as the ratio between AUC_0−24h_ of dihydroartemisinin and artesunate. The geometric mean ratio (GMR: the ratio of the value of the parameter for the drug when used in combination vs the corresponding value for the drug used alone) and its 90 % CI were determined for each parameter. A clinically significant pharmacokinetic drug interaction occurred whenever the 90 % CI for systemic exposure ratio fell entirely outside the equivalence range of 0.8–1.25 [36].

### Statistical analysis

Statistical analysis of the data was performed using SPSS version 16.0 (Gorichem, The Netherlands). Pharmacokinetic parameters are presented as median and 95 % confidence intervals (95 % CI). Comparison of all pharmacokinetic parameters of artesunate, dihydroartemisinin, mefloquine, lopinavir, and ritonavir obtained during the two Periods (−1 vs −3 and −2 vs −3) were performed using Wilcoxon Signed-Rank test. Comparison of the frequency of subjects with adverse events between the two groups was performed using a Chi-square test. Statistical significance level was set at a = 0.05 for all tests.

## Results

### Subject characteristics

Sixteen healthy subjects (eight males and eight females) were included in the pharmacokinetic data analysis; one female subject withdrew from the study before Period 2 due to a positive urine pregnancy test. The median (95 % CI) of age and body weight in male *vs* female subjects was 31 (20–40) vs 29 (21–39) years and 60 (56–62) vs 49 (46–53) kg, respectively. All were healthy as verified by results of clinical, ECG and laboratory investigations (Table 1).

**Table 1:** Clinical and laboratory data at baseline

	Median (95 % CI)
White blood cell count (×10^−3^/µL)	9.65 (7.651–10.5)
RBC blood cell count (×10^−6^/µL)	4.58 (3.99–5.23)
Hematocrit (%)	41.3 (35.6–45.2)
Hemoglobin (g/dL)	13.9 (11.6–14.9)
Platelet count (×10^−3^/µL)	2.42 (2.10–2.70)
BUN (mg/dL)	11.5 (8.9–14.4)
Creatinine (mg/dL)	0.90 (0.70–1.00)
AST (U/L)	18 (16–28)
ALT (U/L)	17 (14–21)
Total protein (g/dL)	7.00 (6.90–7.20)
Albumin (g/dL)	4.40 (4.30–4.50)
Triglyceride (mg/dL)	71 (63–119)
Fasted blood sugar (mg/dL)	91 (81–94)

Data are presented as median (95 % CI) values from 16 subjects

### Pharmacokinetics of artesunate and dihydroartemisinin with and without lopinavir/ritonavir

**Fig. 2:**
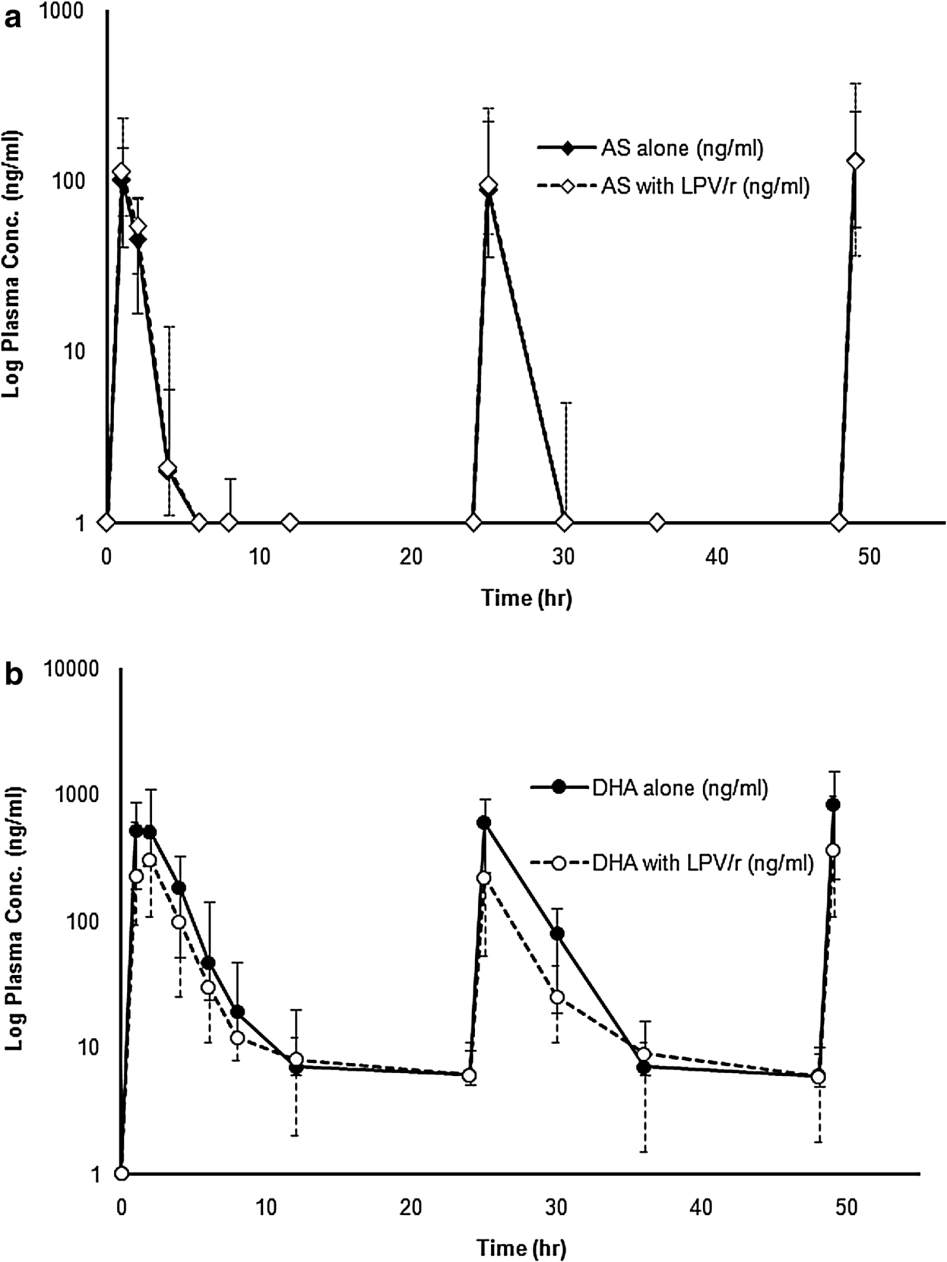
Median (95 % CI) plasma concentration–time profiles of (**a**) artesunate and (**b**) dihydroartemisinin following administrations of a 3-day artesunate-mefloquine, with or without steady-state oral doses of LPV/r

Median (95 % CI) plasma concentration–time profiles of artesunate and dihydroartemisinin following administration of a 3-day artesunate-mefloquine alone (Period 1) and in combination with LPV/r (Period 3) are shown in Fig. 2a, b). None had pre-dose level of either artesunate or dihydroartemisinin on any occasion. The extrapolated AUC of dihydroartemisinin from the last blood sampling time to infinity (AUC_t−∞_) was less than 5 %. The pharmacokinetic parameters of artesunate and dihydroartemisinin are summarized in Table 2. Inter-individual variation of artesunate and dihydroartemisinin plasma concentrations and pharmacokinetic parameters (% CV) ranged from 69 to 400 and 64 to 289.6 %, respectively. Dihydroartemisinin plasma concentrations 1 h post-dose on Day 3 was significantly lower when artesunate was given with LPV/r [median (95 % CI) 310 vs 580 ng/mL, *p* = 0.044]. AUC_0−∞,_ t_1/2z_, CL/F and V_z_/F of artesunate could not be determined due to the rapid elimination of artesunate from the systemic circulation. High inter-individual variability in AUC_0−24h_ for artesunate (54.4 %) and dihydroartemisinin (80.2 %) were observed. In the presence of steady-state LPV/r concentrations, artesunate AUC_0–24h_ was significantly increased by about 80 % (*p* = 0.034), dihydroartemisinin C_max,_ AUC_0−24h_ and AUC_0−∞_ were significantly decreased by about 46.6, 58.2 and 48.8 %, respectively (*p* = 0.023, 0.002, and 0.006, respectively), while t_1/2z_ was significantly increased by 148.5 % (*p* = 0.028). In addition, the metabolic ratio (AUC_0–24h_ ratio) of dihydroartemisinin to artesunate was significantly reduced by 72.2 % (*p* = 0.001).

**Table 2:** Pharmacokinetic parameters of artesunate and dihydroartemisininalone and in combination with LPV/r (*n* = 16)

Pharmacokinetic parameter	Artesunate				Dihydroartemisinin			
	Alone	With LPV/r	GMR (90 % CI)	*p* value	Alone	With LPV/r	GMR (90 % CI)	*p* value
t_max_ (h)	1.00 (1.00, 1.00)	1.00 (0.57, 1.43)	1.09 (0.91, 1.31)	0.317	2.00 (1.57, 2.43)	2.00 (1.57, 2.43)	0.96 (0.74, 1.23)	0.803
C_max_ (ng/mL)	110 (80, 140)	140 (80, 190)	1.59 (1.03, 2.47)	0.056	580 (290, 870)	310 (80, 540)	0.63 (0.38, 1.06)	0.023*
AUC_0–24h_ (ng h/mL)	200 (160, 230)	240 (190, 290)	1.52 (1.09, 2.10)	0.034*	2370 (1450, 3290)	990 (390, 1590)	0.51 (0.31, 0.85)	0.002*
AUC_0–∞_ (ng h/mL)	–	–	–	–	2680 (1270, 4080)	1370 (620, 2120)	0.55 (0.33, 0.90)	0.006*
t_1/2z_ (h)	–	–	–	–	4.02 (1.52, 6.51)	9.99 (3.43, 16.55)	1.89 (1.00, 3.58)	0.028*
CL/F (L/h)	–	–	–	–	–	–	–	1.00
V_z_/F (L/kg)	–	–	–	–	–	–	–	1.00
Metabolic ratio	11.78 (7.56, 15.99)	3.27 (1.62, 4.92)	0.23 (0.11, 0.47)	0.001*				

Data are presented as median (95 % CI) or GMR (90 % CI)

* Statistically significant difference (Wilcoxon Signed-Rank test)

### Pharmacokinetics of mefloquine with and without lopinavir/ritonavir

**Fig. 3:**
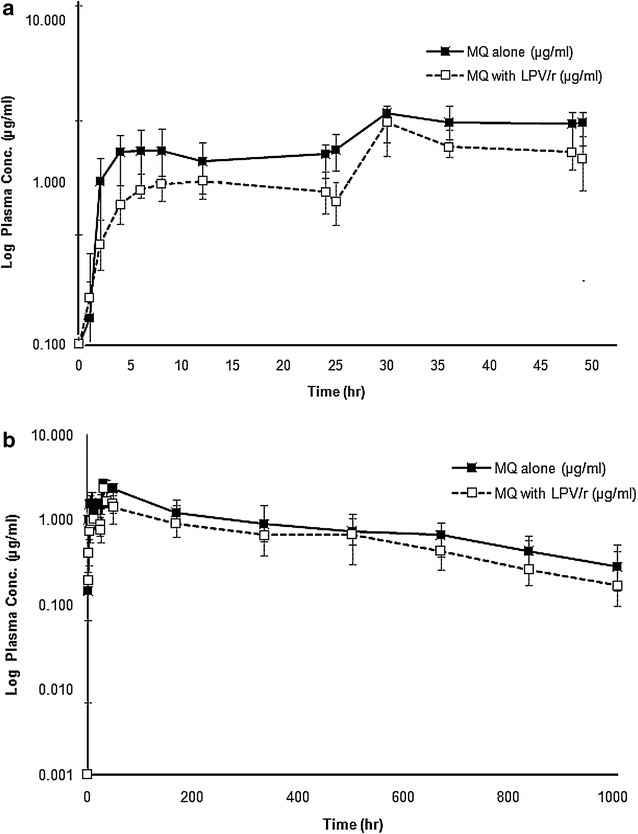
Median (95 % CI) plasma blood concentration–time profiles of mefloquine following administrations of a 3-day artesunate-mefloquine during (**a**) 48 h and (**b**) 42 days, with or without steady-state oral doses of LPV/r

Median (95 % CI) plasma concentration–time profiles of mefloquine following administration of a 3-day artesunate-mefloquine alone (Period 1) and in combination with LPV/r (Period 3) are shown in Fig. 3a, b, and its pharmacokinetic parameters are summarized in Table 3. None had pre-dose level of mefloquine on any occasion. The extrapolated AUC of mefloquine from the last blood sampling time to infinity (AUC_t−∞_) was less than 10 %. In the presence of LPV/r, mefloquine C_max_, AUC_0−48h_, AUC_0−168h_ and AUC_0−∞_ were significantly decreased by 19.3, 28.7, 37.1 and 35.2 %, respectively (*p* = 0.039, 0.001, 0.002 and 0.007, respectively), while V_z_/F and CL/F were significantly increased by 37.9 and 54.6 %, respectively (*p* = 0.004 and 0.010, respectively). The inter-individual variability of plasma mefloquine concentrations ranged from 34.2 to 96.9 %.

**Table 3:** Pharmacokinetic parameters of mefloquine when administered alone and in combination with LPV/r

Pharmacokinetic parameter	Mefloquine			*p* value
	Alone	With LPV/r	GMR (90 % CI)	
t_max_ (h)	33.0 (30.4, 35.5)	33.0 (30.4, 35.5)	1.04 (0.94, 1.14)	0.473
C_max_ (ng/mL)	2900 (2440, 3350)	2340 (2050, 2630)	0.87 (0.72, 1.06)	0.039*
AUC_0–48h_ (μg h/mL)	952 (711, 1192)	678 (474, 882)	0.72 (0.54, 0.97)	0.001*
AUC_0-168_ (μg h/L)	331 (283, 380)	208 (169, 248)	0.70 (0.55, 0.88)	0.002*
AUC_0–∞_ (μg h/mL)	1160 (924, 1397)	751 (471, 1031)	0.75 (0.54, 1.04)	0.007*
t_1/2z_ (h)	344 (284, 404)	336 (267, 406)	1.14 (0.90, 1.45)	0.408
CL/F (L/h)	1.08 (0.78, 1.37)	1.67 (1.03, 2.30)	1.33 (0.96, 1.84)	0.004*
V_z_/F (L/kg)	9.24 (7.67, 10.81)	12.75 (8.15, 17.35)	1.51 (1.10, 2.08)	0.010*

Data are presented as median (95 % CI) and GMR (90 % CI)

* Statistically significant difference (Wilcoxon Signed-Rank test)

### Pharmacokinetics of lopinavir/ritonavir with and without artesunate-mefloquine

**Fig. 4:**
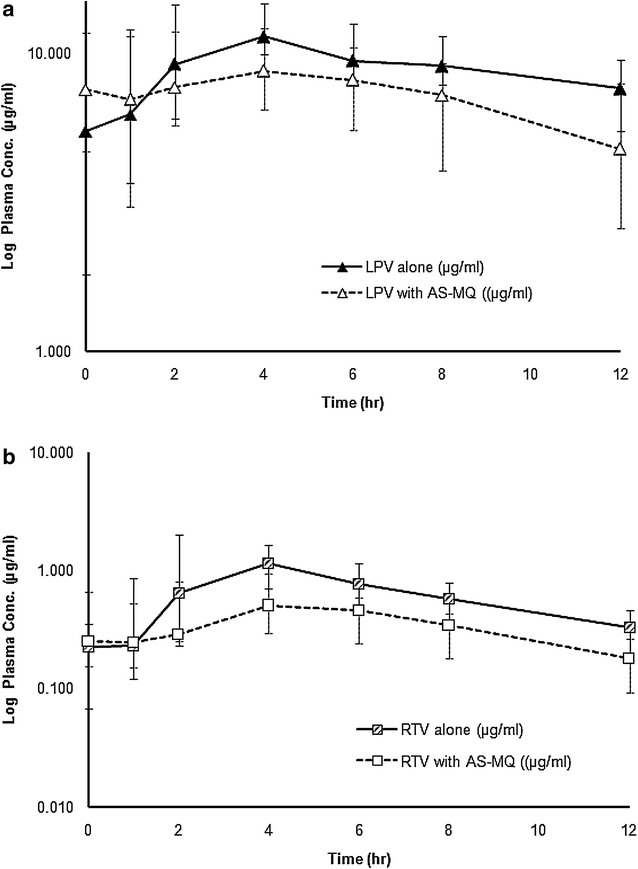
Median (95 % CI) plasma concentration–time profiles of (**a**) lopinavir and (**b**) ritonavir following oral doses of 400 mg lopinavir plus 100 mg ritonavir twice a day, with or without a 3-day artesunate-mefloquine

Median (95 % CI) plasma concentration–time profiles of LPV/r following the administration during Period 2 and 3 are shown in Fig. 4a, b. None had pre-dose level of lopinavir or ritonavir on any occasion. Three subjects in Period 1 and one subject in Period 3 had undetectable plasma lopinavir concentrations until 1 h after the first dose. One subject during Periods 1 and 3 each had undetectable plasma lopinavir concentrations until 1 h after the first dose in Periods 1 and 3. The extrapolated AUC of lopinavir or ritonavir from the last blood sampling time to infinity (AUC_t−∞_) was less than 5 %. The inter-individual variability of plasma lopinavir and ritonavir concentrations ranged from 50.6 to 127.6 and 21.3 to 62.9 %, respectively.

The pharmacokinetics of lopinavir and ritonavir alone (Period 2) and in combination with artesunate-mefloquine (Period 3) are summarized in Table 4. In the presence of artesunate-mefloquine, the C_max_ of lopinavir was significantly decreased by 22.1 % (*p* = 0.03), while CL/F was significantly increased by 75.4 % (*p* = 0.023). The AUC_0–12h_, AUC_0−∞_ and C_max_, of ritonavir were significantly decreased by 44.6, 56.3 and 54.9 %, respectively (*p* = 0.003, 0.010, and 0.004, respectively), while CL/F and V_z_/F were significantly increased by 129.1 and 80.4 %, respectively (*p* = 0.008 and 0.04, respectively).

**Table 4:** Pharmacokinetic parameters of ritonavir and lopinavir when administered as LPV/r alone and in combination with artesunate-mefloquine (*n* = 16)

Pharmacokinetic parameter	Lopinavir				Ritonavi			
	Alone	With artesunate-mefloquine	GMR (90 % CI)	*p* value	Alone	With artesunate-mefloquine	GMR (90 % CI)	*p* value
t_max_(h)	4.0 (3.1, 4.8)	4.0 (3.1, 4.8)	0.86 (0.63, 1.17)	0.887	4.0 (3.1, 4.8)	4.0 (3.1, 4.8)	1.03 (0.75, 1.43)	0.942
C_max_ (μg/mL)	12.36 (10.51, 14.21)	9.62 (7.99, 11.24)	0.77 (0.63, 0.92)	0.030*	1.53 (0.97, 2.08)	0.68 (0.49, 0.87)	0.48 (0.34, 0.69)	0.004*
AUC_0–12h_ (μg h/mL)	103.32 (82.62, 124.01)	90.51 (74.86, 106.16)	0.78 (0.62, 0.99)	0.088	8.11 (5.71, 10.50)	4.49 (3.47, 5.50)	0.52 (0.37, 0.74)	0.003*
AUC_0–∞_ (μg h/mL)	228.98 (131.13, 326.82)	130.19 (22.61, 237.76)	0.62 (0.38, 1.03)	0.099	10.79 (6.17, 15.40)	4.71 (2.27, 7.15)	0.46 (0.29, 0.71)	0.010*
t_1/2z_ (h)	9.8 (5.4, 14.2)	6.6 (1.8, 11.4)	0.71 (0.45, 1.13)	0.071	4.3 (3.5, 5.1)	3.7 (3.1, 4.4)	0.87 (0.68, 1.11)	0.239
CL/F (L/h)	1.75 (1.01, 2.49)	3.07 (1.18, 4.96)	1.61 (0.98, 2.63)	0.023*	9.27 (4.74, 13.80)	21.24 (8.71, 33.77)	2.19 (1.41, 3.40)	0.008*
V_z_/F (L/kg)	0.45 (0.36, 0.54)	0.53 (0.41, 0.65)	1.17 (0.99, 1.38)	0.071	0.97 (0.61, 1.32)	1.75 (0.13, 3.37)	1.90 (1.29, 2.81)	0.041*

Data are presented as median (95 % CI) or GMR (90 % CI)

* Statistically significant difference (Wilcoxon Signed-Rank test)

### Pharmacokinetic interaction between a 3-day artesunate-mefloquine and lopinavir/ritonavir

The 90 % CI of the GMR of C_max_ for dihydrartemisinin, lopinavir, ritonavir, and mefloquine, AUC_0−12h_ for ritonavir, AUC_0−24h_ for artesunate and dihydroartemisinin, AUC_0−168h_ for mefloquine, and AUC_0−∞_ for mefloquine and ritonavir were outside the acceptable bioequivalent range of 0.8-1.25 (Tables 2, 3 and 4).

### Safety and tolerability

Drug treatments were generally well tolerated. Only mild to moderate (NIH/NCI Grade 1 and 2) severity grade adverse events possibly related to the study drug(s) was observed. The frequency of adverse events occurred during the three periods were similar. The adverse events during Period 1 (artesunate-mefloquine combination) included vertigo (11 cases, 68.75 %), and nausea/vomiting (eight cases, 50 %). The adverse events observed during Period 2 (LPV/r) were diarrhoea (eight cases, 50 %) and vertigo (one case, 6.25 %). The adverse events observed during Period 3 (artesunate-mefloquine plus LPV/r) were vertigo (eight cases, 50 %), nausea/vomiting (two cases, 12.5 %), poor appetite (two cases, 12.5 %), and syncope (one case, 6.25 %). A markedly high proportion of subjects (11/16) with increased serum triglyceride (about 0.5–2.5 times of baseline) was observed during Period 2 after 28 doses of LPV/r.

## Discussion

The present study is the first reporting the pharmacokinetic interactions between the artesunate-mefloquine and LPV/r. Marked changes in the pharmacokinetics of artesunate, dihydroartemisinin, mefloquine, lopinavir, and ritonavir were observed. All dose regimens during the three periods were relatively well tolerated with no serious adverse events. Vertigo, nausea and vomiting were the most common adverse event during artesunate-mefloquine in this study. The adverse events occurred during administration of LPV/r, diarrhoea and vertigo, were similar to previous observations [37–42]. Protease inhibitors have been commonly associated with the elevation of triglyceride in all age groups of HIV-infected patients [43, 44].

The disposition kinetics of artesunate and dihydroartemisinin were in general agreement with that observed in healthy Thai subjects [45]. Due to rapid clearance of artesunate from the systemic circulation, estimation of CL/F, V_z_/F, and t_1/2z_ were not possible. In addition, estimation of AUC_0−∞_ of artesunate is not accurate due to a large inter-individual variability of artesunate concentrations at the last sampling time point. A relative longer t_1/2z_ of dihydroartemisinin was noted compared with a previous report (4.02 vs 0.74 h) [46]. Multiple factors complicate comparison of artesunate/dihydroarteminin pharmacokinetic findings across studies, including differences in assay sensitivities and blood sampling schedules. Additionally, the pharmacology of these agents is known to be different between patients with acute malaria and healthy volunteers. Mefloquine, a fluorinated 4-quinoline methanol compound, is moderately well absorbed orally in this split-dose regimen and extensively distributed. Its elimination was best described by biexponential disposition kinetics. The systemic exposure of mefloquine given as two divided doses of 750 and 500 mg 24 h apart appeared to be higher, i.e., ~two-fold relative to that observed in the previous study in healthy Thai subjects following mefloquine alone given at 750 and 500 mg 6 h apart [47, 48]. This was attributed to a relatively smaller V_z_/F (40 %) and lower CL/F (33.4 %) in the current study population. Previous studies have shown that different mefloquine products are not bio-equivalent [49, 50]. Furthermore, mefloquine concentrations in plasma and whole blood have been shown to be different [51, 52]. Direct comparison of pharmacokinetics particularly systemic exposure should therefore be made with caution. The pharmacokinetics of lopinavir and ritonavir were in agreement with published data in HIV-negative volunteers [53].

Two studies have reported the pharmacokinetic dug-drug interactions between artesunate-based combinations with antiretrovirals drug. In the presence of steady-state nevirapine-based HIV-antiviral therapy, AUC_0−∞_ of artesunate was increased 1.5-fold, while V_z_/F and CL/F were decreased [54]. In contrast, steady-state ritonavir concentrations significantly reduced the AUC_0−∞_ and C_max_ of dihydroartemisinin in healthy subjects [55]. It was thought that the influence of mefloquine on pharmacokinetics of artesunate/dihydroartemisinin would only be minimal, if any [47]. The apparent decrease in systemic exposure of mefloquine in the presence of LPV/r could not be explained by the inhibitory effect of LPV/r on CYP3A4-mediated mefloquine metabolism [56]. Ritonavir was found to minimally affect mefloquine pharmacokinetics despite its strong inhibitory activity on CYP3A4 following a single 200 mg dose in healthy volunteers [56]. On the other hand, ketoconazole, the CYP3A4 inhibitor was found to increase plasma mefloquine concentrations in healthy subjects when co-administered at the dose of 400 mg daily for 10 days [57].

Based on available information, mefloquine does not interact with many compounds, although in vitro data suggested that it is a substrate and inhibitor of CYP3A4 and P-glycoprotein [17, 58, 59] and animal data indicated that it reduces bile production in rats [60]. Since both mefloquine and LPV/r are substrates of CYP3A4, the decrease in systemic exposure together with an increase in total oral clearance without a change in t_1/2z_ of mefloquine may suggest the possibility of an inducing effect of LPV/r on the intestinal P-glycoprotein [61, 62] and/or a decrease in intestinal absorption of mefloquine. The increases in CL/F and Vz/F of mefloquine are of similar magnitude, whereas t_1/2z_ is unchanged. This supports a decrease in mefloquine bioavailability due to LPV/r. The significant decrease in mefloquine C_max_ and the similarity in magnitude of the increases in CL/F and Vz/F of mefloquine without change in t_1/2z_ may also support this supposition.

The decrease in systemic exposure of both lopinavir and ritonavir was also unexpected in the light of report on the inhibitory effect of mefloquine on CYP3A4 [17]. Ritonavir auto-induction would be expected to be relatively low, but a decrease in intestinal absorption as a consequence of inhibitory effect of both mefloquine and ritonavir on bile production is possible [23, 60, 63]. Furthermore, because ritonavir is a substrate of P-glycoprotein [64], induction of gut P-glycoprotein by mefloquine and/or carboxymefloquine may contribute to the decreased drug absorption. In a previous study [56], ritonavir steady-state AUC was significantly decreased by mefloquine without any change in t_1/2z_ following multiple dosing.

The systemic exposure of artesunate and dihydroartemisinin were changed in opposite direction in the presence of LPV/r (increased artesunate and decreased dihydroartemisinin exposure), and thus did not support the inducing activity of carboxymefloquine on CYP2A6-mediated artesunate metabolism [15]. The decrease in dihydroartemisinin AUC0-24 h when expressed as nmol h/mL unit is much greater than (about 180 %) the increase in artesunate AUC which stronger support on induction of DHA clearance by LOP/r. The contribution of mefloquine on disposition of artesunate/dihydroartemisinin was unlikely [47]. In addition, it was noted however that the term ‘metabolic ratio’ may not be the best to describe the rate of disappearance of artesunate in plasma as it may be contributed to by transport proteins. It should be pointed out that the observed systemic exposure of dihydroartemisinin may reflect both the absorption of artesunate, with subsequent conversion to dihydroartemisinin through first-pass or systemic metabolism, as well as direct absorption of dihydroartemisinin following its formation in the gut through acid-dependent chemical hydrolysis [65].

A large reduction in systemic exposure of both the anti-malarial artesunate-mefloquine and the antiretroviral LPV/r raises concerns regarding the higher risk of treatment failure of both malaria and HIV infection when artesunate-mefloquine and LPV/r are co-administered. The influence of disease factors (both malaria and HIV) add more complexity on the interaction [18, 66–68]. On the parasitological aspect, in vitro studies suggest that antiretroviral protease inhibitors may also possess anti-malarial activity [69–72] and could potentiate the efficacy of anti-malarial drugs [73–75]. Combination of mefloquine with ritonavir was shown to produce synergistic anti-malarial activity against D10 and FAC8 *P. falciparum* clones in vitro [69]. The most concerning finding is a 50 % reduction in the AUC of dihydroartemisinin and a significant decrease in Day 7 exposure of mefloquine. Artesunate and dihydroartemisinin (three- to four-fold potency of artesunate) play an important role the first phase of malaria therapy by rapidly lowering the parasite burden. Therefore, any decrease in dihydroartemisinin exposure as a consequence LPV/r co-administration may increase the risk of delayed parasite clearance, particularly given recent evidence of resistance to the artemisinins emerging in Southeast Asia [76]. Current guidelines from the WHO for treatment of uncomplicated malaria emphasize the need for 3 days of adequate ACT exposure to ensure elimination of parasites [13, 77]. Given that HIV/malaria co-infected patients present with higher parasite counts [8, 78], any reduction in dihydroartemisinin exposure may predispose patients to develop severe malaria due to slower parasite clearance. On the other hand, the reduction in systemic clearance of mefloquine would increase the risk of parasite recrudescence and/or re-infection. The Day 7 whole blood mefloquine concentration has proved to be a useful and simple surrogate for therapeutic efficacy of mefloquine [79].

## Conclusion

Significant changes in the pharmacokinetics of artesunate/dihydroartemisinin, mefloquine and LPV/r were observed in the current study. Despite some limitations of the studies (small sample size, no measurement of carboxymefloquine and free drug concentrations), these data do provide valuable insights into the potential pharmacokinetic interactions when artesunate-mefloquine is co-administered with LPV/r in adults. The complex interaction unexplained by the metabolic behaviour (both parent drugs and metabolites) may suggest the involvement of multiple drug metabolizing enzymes and drug transporters. Studies in the target population (HIV and malaria co-infection) are needed for more conclusive clinical relevance and mechanistic explanation. Until this information is available, extrapolating these findings to the target population should be performed with caution.
